# Association Between Waste-Free Formularies and Prescription Drug Spending Among Self-insured Employers

**DOI:** 10.1001/jamanetworkopen.2021.31486

**Published:** 2021-10-28

**Authors:** Mariana P. Socal, Ge Bai, Thomas Cordeiro, Gerard F. Anderson

**Affiliations:** 1 Johns Hopkins Bloomberg School of Public Health, Baltimore, Maryland; 2 Johns Hopkins Carey Business School, Baltimore, Maryland; 3 Integrity Pharmaceutical Advisors, North Charleston, South Carolina

## Abstract

This quality improvement study evaluates the association between waste-free formularies and prescription drug spending for 2 large self-insured employers.

## Introduction

Wasteful prescription drug spending arises when drugs are therapeutically appropriate but cost substantially more for the same clinical benefit as their therapeutic alternatives.^1^ Because these drugs and their alternatives (eg, an expensive proprietary product and its less expensive generic equivalent) have the same clinical outcomes, wasteful drug spending differs categorically from unnecessary or inappropriate use.^2,3^ Recently, some self-insured employers have implemented waste-free formularies to contain drug spending, but the results have not yet been documented in the literature.^4,5^

This study examines the experience of 2 large self-insured employers that shared per-member per-month (PMPM) spending information, net of rebates and discounts, before and after the implementation of their waste-free formularies. Employer 1, a large public employer, spends approximately $200 million annually on prescription drugs for more than 300 000 beneficiaries. Employer 2, a publicly traded Fortune 500 company, spends approximately $100 million annually on prescription drugs for more than 60 000 beneficiaries.

## Methods

Both employers hired external pharmacy consultants to perform a drug utilization review, identify wasteful drugs, and implement appropriate substitutions. Employer 1 gradually modified its formulary between January 2017 and December 2018, and employer 2 implemented its waste-free formulary in January 2019. This quality improvement study compared the following before and after waste-free formulary implementation: (1) average prerebate 30-day drug spending for the therapeutic classes targeted and not targeted by the waste-free formularies and (2) annual postrebate PMPM spending for all therapeutic classes.

This study was exempt from institutional review board approval because it did not meet criteria for human subjects research, in accordance with the Common Rule. The study followed the Standards for Quality Improvement Reporting Excellence (SQUIRE) reporting guideline.

## Results

The 2 employers identified approximately 300 drugs as potentially wasteful. For employer 1, these drugs represented 10% of all drugs covered in its formulary, 9% of the covered therapeutic classes, and 29% of all pharmacy claims. For employer 2, these drugs represented 14% of all drugs covered in its formulary, 3% of the covered therapeutic classes, and 17% of all pharmacy claims.

Most potentially wasteful drugs (279 of 293 [95%]) were excluded from the original formularies and replaced with less expensive therapeutic alternatives of the same clinical value. The 14 remaining drugs (5%) were placed under prior authorization or step-therapy requirements. Substituted drugs belonged to 3 categories: multisource products (76 [26%]), me-too products (118 [40%]), and same-class products (85 [29%]) (Table). All 14 drugs placed under prior authorization or step-therapy requirements were same-class products. As a result, the average prerebate 30-day spending across all drugs in the therapeutic classes targeted by the waste-free formularies declined by 53% for employer 1 (the utilization rate of these drugs increased by 6%) and 67% for employer 2 (the utilization rate of these drugs decreased by 19%) (Figure). Prerebate 30-day spending on nontargeted classes decreased by 0.5% and 6%, respectively, in this same period. The annual postrebate PMPM, across all drugs covered on the formulary (including specialty drugs) and net of all rebates and discounts, decreased by 9% and 15%, respectively.

**Table.** Characteristics of Potentially Wasteful Drugs

Category	Description	No. (%) of all wasteful drugs	Example of wasteful drug (unit price, $)	Example of therapeutic alternative (unit price, $)
Multisource products	The wasteful product (usually proprietary) and the alternative cheaper product (usually generic) have the same active ingredient, dosage form, and strength	76 (26)	Sumatriptan (Imitrex), 100 mg (80.73)	Generic sumatriptan, 100 mg (25.14)
Me-too products	The wasteful product has minimal differences compared with the cheaper alternative, but no major difference in clinical effectiveness	118 (40)		
Different chemical formulation	Different salt or isomer form	11 (4)	Generic esomeprazole magnesium, 20 mg (1.02-9.02)	Generic omeprazole magnesium, 20 mg (0.59-0.77)
Different dosage form	Capsule vs tablet	52 (18)	Diclofenac (Zipsor) capsules, 25 mg (9.36)	Generic diclofenac EC tablets, 25 mg (1.42)
Different strength	2 Pills of 20 mg vs 1 pill of 40 mg	40 (14)	Fenofibrate (Lipofen) capsules, 150 mg (9.54)	Generic fenofibrate tablets, 160 mg (0.61-2.88)
Fixed-dose combinations (“combo drugs”)	The combination of wasteful products is significantly more expensive than the components taken separately	15 (5)	Naproxen + sumatriptan (Treximent) tablets, 85 + 500 mg (155.75)	Sumatriptan, 100 mg (1.30-25.14) + naproxen EC tablet, 500 mg (8.41)
Same-class products	The wasteful product has a cheaper alternative that is a different drug in the same therapeutic class with the same mechanism of action and equivalent clinical effectiveness and safety	85 (29)	Rabeprazole (Aciphex) oral EC tablets, 20 mg (20.99)	Generic omeprazole magnesium, 20 mg (0.59-0.77)

Abbreviation: EC, enteric coated.

These data reflect 279 of a total of 293 drugs from 51 therapeutic classes that were substituted as a result of the waste-free formularies, pooled across the 2 self-insured employers. The 14 drugs subjected to step-therapy or preauthorization requirements were not included on this table because they were not substituted with other products. Drugs were identified by their proprietary name. Substitutions were counted only once per drug and type; that is, if 3 strengths of the same drug were substituted under the waste-free formularies, the drug was included in the “different strength” category only once. Price information was obtained from the Lexicomp online database, reflecting the average wholesale price (AWP) or prerebate price (last updated on January 2, 2021; data collected on January 8, 2021). A range was listed when more than 1 manufacturer’s AWP was available. Percentages may not add to 100% because of rounding.

**Figure.**
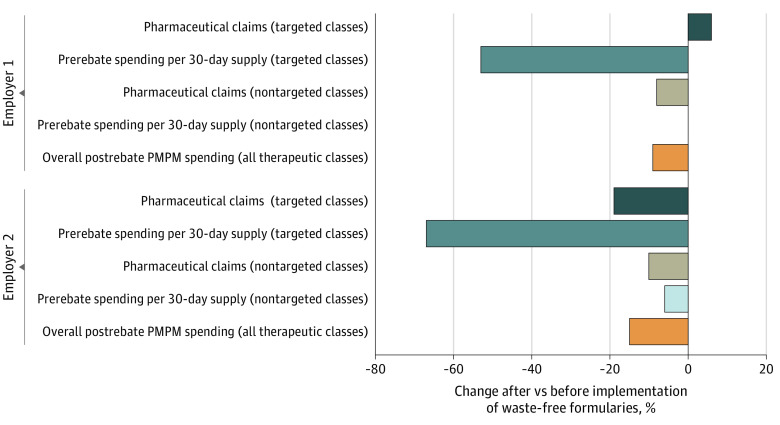
Prescription Drug Utilization Rates and Spending Levels for 2 Self-insured Employers After vs Before the Implementation of Waste-Free Formularies Pharmaceutical claims and prerebate spending per 30-day supply are averages presented separately for therapeutic areas targeted by the waste-free formularies and for therapeutic areas not targeted by the waste-free formularies. Overall postrebate per-member per-month (PMPM) spending reflects the pharmacy spending, net of all rebates and discounts, across all drugs and therapeutic classes covered in the employer’s formulary (targeted and nontargeted classes). “Before” and “after” periods were defined as 2 quarters before and 2 quarters after the implementation of the waste-free formularies, respectively.

Both employers notified beneficiaries who were affected by prescription drug coverage changes 90 days in advance. Employer 2 hired pharmacists to help beneficiaries navigate the transition. During the implementation phase, employer 1 received slightly higher-than-normal beneficiary complaints, whereas employer 2 did not.

## Discussion

For the 2 large employers in this study, potentially wasteful drugs constituted a small percentage of all covered drugs (10%-14%) and were concentrated in a few therapeutic classes (3%-9%), but they represented a significant proportion of pharmaceutical claims (17%-29%). Within the targeted therapeutic classes, the prerebate savings surpassed 50%. Overall postrebate PMPM savings were 9% to 15%. To our knowledge, these results provide the first systematic evidence on the cost-saving potential of implementing waste-free formularies. Although approximately 300 drugs were identified as potentially wasteful between the 2 employers in this study, many more such drugs exist. As drug prices fluctuate and new drugs enter the market, the wasteful spending potential of covered drugs should be reassessed periodically.

The finding that the most common substitution among potentially wasteful drugs in the waste-free formulary was me-too drugs (118 [40%]), followed by same-class drugs (85 [29%]), suggests that cost-saving opportunities go beyond generic substitutions of multisource drugs. With effective communication, implementing waste-free formularies may not generate beneficiary dissatisfaction.^6^ Public programs and private insurance plans interested in reducing wasteful prescription drug spending should consider implementing waste-free formularies, facilitated by effective communication and navigation efforts to ensure beneficiary satisfaction. The results of this study, limited by the experience of 2 large employers, may not be generalizable to small employers. Future studies examining clinical outcomes associated with the transition to waste-free formularies are warranted.

## References

[1] Organization for Economic Cooperation and Development. Tackling wasteful spending on health. January 10, 2017. Accessed May 21, 2021. https://www.oecd.org/health/tackling-wasteful-spending-on-health-9789264266414-en.htm

[2] Shrank WH, Rogstad TL, Parekh N. Waste in the US health care system: estimated costs and potential for savings. JAMA. 2019;322(15):1501-1509. doi:10.1001/jama.2019.13978 31589283

[3] Office of the Assistant Secretary for Planning and Evaluation, US Department of Health and Human Services. Savings available under full generic substitution of multiple source brand drugs in Medicare Part D. July 23, 2018. Accessed May 21, 2021. https://aspe.hhs.gov/sites/default/files/private/pdf/259326/DP-Multisource-Brands-in-Part-D.pdf

[4] Vela L. Reducing wasteful spending in employers’ pharmacy benefit plans. The Commonwealth Fund. August 30, 2019. Accessed May 21, 2021. https://www.commonwealthfund.org/publications/issue-briefs/2019/aug/reducing-wasteful-spending-employers-pharmacy-benefit-plans

[5] Midwest Business Group on Health. Employer journey—prescription drug benefits redesign: using common sense to promote value for all stakeholders. Accessed May 21, 2021. http://www.specialtyrxtoolkit.org/managing-your-strategy/benefit-strategies-plan-design/employer-case-study-pharmacy-benefit-redesign/

[6] Anderson GF, Cordeiro TC, Socal MP, Vela L. Removing waste from drug formularies: a practical guide to help employers remove waste from drug formularies and achieve savings for companies and employees while maintaining member satisfaction. Accessed May 21, 2021. https://www.pbgh.org/wp-content/uploads/2021/01/PBGH-Wasteful-Drugs-Guidebook-FINAL.pdf

